# Investigation of Linear Amplification Using Abasic Site-Containing Primers Coupled to Routine STR Typing for LT-DNA Analysis

**DOI:** 10.3390/genes13081386

**Published:** 2022-08-04

**Authors:** Xiaoqin Qian, Zhimin Li, Zhihan Zhou, Jinglei Qian, Yining Yao, Chengchen Shao, Qiqun Tang, Jianhui Xie

**Affiliations:** 1 Department of Forensic Medicine, School of Basic Medical Sciences, Fudan University, Shanghai 200032, China; 2 Department of Biochemistry and Molecular Biology, School of Basic Medical Sciences, Fudan University, Shanghai 200032, China

**Keywords:** low-template DNA, linear amplification, abasic site, DNA polymerases, short tandem repeats

## Abstract

Obtaining a full short tandem repeat (STR) profile from a low template DNA (LT-DNA) still presents a challenge for conventional methods due to significant stochastic effects and polymerase slippage. A novel amplification method with a lower cost and higher accuracy is required to improve the DNA amount. Previous studies suggested that DNA polymerases without bypass activity could not perform processive DNA synthesis beyond abasic sites in vitro and our results showed a lack of bypass activity for Phusion, Pfu and KAPA DNA polymerases in this study. Based on this feature, we developed a novel linear amplification method, termed Linear Aamplification for double-stranded DNA using primers with abasic sites near 3′ end (abLAFD), to limit the replication error. The amplification efficiency was evaluated by qPCR analysis with a result of approximately a 130-fold increase in target DNA. In a LT-DNA analysis, the abLAFD method can be employed as a pre-PCR. Similar to nested PCRs, primer sets used for the abLAFD method were designed as external primers suitable for commercial multiplex STR amplification assays. The practical performance of the abLAFD method was evaluated by coupling it to a routine PP21 STR analysis using 50 pg and 25 pg DNA. Compared to reference profiles, all abLAFD profiles showed significantly recovered alleles, increased average peak height and heterozygote balance with a comparable stutter ratio. Altogether, our results support the theory that the abLAFD method is a promising strategy coupled to STR typing for forensic LT-DNA analysis.

## 1. Introduction

Low-template DNA (LT-DNA) is commonly encountered in forensic cases, such as touched DNA [1,2,3,4]. It is a great challenge to obtain full short tandem repeat (STR) profiles from a LT-DNA [5,6]. There are two main factors impairing the STR typing for a LT-DNA including stochastic effects and polymerase slippage. Stochastic effects could result in a low reproducibility of STR profiles, and the polymerase slippage could result in the accumulation of PCR artifacts due to exponential amplification. Several strategies were adopted to improve the efficiency of STR typing from the LT-DNA, including increasing amplification cycles, reducing the reaction volume, post-amplification purification, and injection enhancement by increased voltage or time [7,8,9,10,11,12]. However, because of a high polymerase slippage rate, the increase in amplification cycles could result in a higher stutter and/or allelic drop-in (ADI). The post-amplification optimization for the detection of STR genotypes was able to improve the recovery of alleles for relatively low allelic products, but not for very low or absent ones.

In another attempt to improve the sensitivity of the STR typing, the preamplification for the LT-DNA to increase the DNA templates for the STR typing was also investigated. Whole genome amplification (WGA) was able to increase the amount of DNA template but lacked the reproducibility due to the binding of random primers and the polymerase slippage [13,14,15]. Conventional nested PCR was able to selectively amplify locus-specific genomic DNA, but concern was raised about the polymerase slippage which may result in ADI due to the exponential amplification [7]. In contrast, locus-specific linear amplification of genomic DNA could reduce the accumulation of PCR artifacts for following the STR typing. Recently, a linear amplification method was developed for LT-DNA analysis by amplifying targets with either forward or reverse primers in a single reaction, which suggests improved allele recovery in a multiplex STR analysis [16]. Since only one strand of double-stranded DNA molecules was amplified in single-primer reactions, half of the LT-DNA samples could not be fully utilized in such a detection assay. 

The apurinic/apyrimidinic site, also known as an abasic site, is one of the most frequent spontaneous lesions in genomic DNA, where the DNA base is released by hydrolysis of the N-glycosidic bond and the phosphodiester backbone is left intact [17]. During DNA replication, DNA polymerases without bypass (translesional) activity can stall at these abasic sites, leading to the termination of replication [18,19]. In a previous study, DNA containing an abasic site as a template was able to create mutation at this locus using translesional Taq DNA polymerase [20]. In contrast, KOD DNA polymerase with 3′-5′ exonuclease activity was unable to amplify the DNA template with an abasic site [21]. 

**Figure 1:**
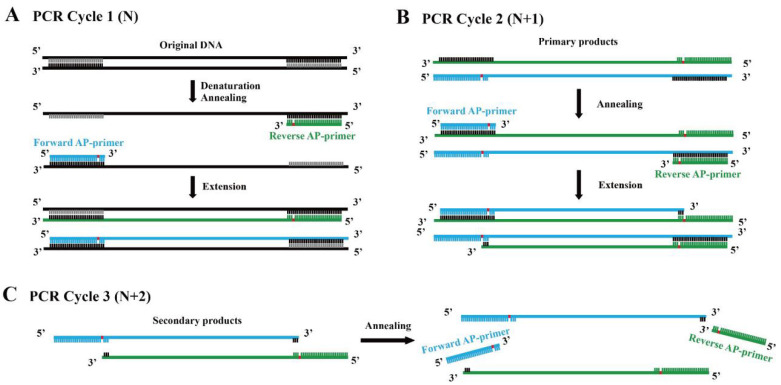
Replication mechanism of the abLAFD method. The location of the abasic site in primers is marked in red. (**A**): In PCR cycle 1 (N), the abasic site is introduced into the primary products. (**B**): In PCR cycle 2 (N + 1), the abasic site in the primary products acts as a blocker for the subsequent dNTP incorporation to the secondary nascent DNA strand, which results in the absence of a primer binding site in the secondary products. (**C**): In PCR cycle 3 (N + 2), abasic site-containing primers cannot hybridize with the secondary products if annealing conditions remain unchanged. Thus, only original DNA template and primary products copied directly from original DNA molecules can serve as templates in the abLAFD reaction, which leads to a linear amplification fashion.

Based on this fact, we assumed that a novel linear amplification could be developed by employing DNA polymerase which lacks bypass activity and primers with an abasic site several nt (empirically 5–9 nt) away from the 3′ end. We termed this method Linear Amplification for double-stranded DNA using primers with abasic sites near 3′ end (abLAFD). Theoretically, the abasic site can be introduced into the primary nascent DNA strand complementarily to the original DNA template in each cycle (Figure 1A). When the primary nascent DNA strand starts to work as a template from the second cycle, the abasic site in the template acts as a blocker for the subsequent dNTP incorporation, which results in the absence of a primer binding site in the secondary nascent DNA strand (Figure 1B). For following PCR cycles, the secondary nascent DNA strands cannot be used as DNA templates under the annealing conditions (Figure 1C). Therefore, only the original DNA template and primary products copied directly from original DNA molecules can serve as templates in this method, which leads to a linear amplification fashion.

To reduce amplification bias and increase the starting template in the LT-DNA analysis, the abLAFD method could be coupled to regular STR analysis. Similar to nested PCR, primer sets used for the abLAFD method could be designed as external primers suitable for commercial multiplex STR amplification assays. 

In this study, we aimed to develop the abLAFD method and verify its feasibility in an LT-DNA analysis. The bypass activity of the DNA polymerase was investigated to identify suitable DNA polymerase for use in the abLAFD method. Then, the amplification efficiency of the abLAFD method as well as the performance of this pre-PCR procedure coupled to STR analysis in the LT-DNA were evaluated. 

## 2. Materials and Methods

### 2.1. Sample, DNA Polymerase and Primer Design

Human female DNA 9947A (NuHigh Biotechnologies Co. Ltd, Suzhou, China) was used as a template in this study. Four DNA polymerases were used, including Taq (Novoprotein Scientific, Suzhou, China) from Family A, and Phusion (Thermo Fisher Scientific, Waltham, MA, USA), KAPA HiFi (Roche, Basel, Switzerland) and Pfu (Beyotime Biotechnology, Shanghai, China) from Family B, together with their respective master mix. The replicative DNA polymerases from Family A are found in bacteria, and those from Family B are found in eukarya and archaea [22]. Taq DNA polymerase is from *Thermous aquaticus* and lacks 3′-5′ exonuclease activity with a high replication error rate (about 2 × 10^−5^). Taq can add a non-template A-tail at the 3′ end of nascent DNA strands, which can be directly used for TA cloning. Pfu DNA polymerase is from *Pyrococcus furiosus*. According to the manufacturers’ guidebooks, Phusion is a novel *Pyrococcus*-like enzyme fused with a processivity-enhancing domain and KAPA is an engineered Family B DNA polymerase without the need for accessory proteins or DNA binding domains. Pfu, Phusion and KAPA DNA pomerase have a functional domain with the proofreading 3′-5′ exonuclease activity which ensures high fidelity of the DNA synthesis. These three polymerases lack non-template A-tail activity and generate blunt-ended products. However, whether these polymerases have the bypass activity at abasic sites remains unknown. Primer sets were designed using Primer 3 software and were synthesized with or without modification (dSpacer, 6-FAM) by Sangon Biotech, China.

### 2.2. The Development of the abLAFD Method

#### 2.2.1. DNA Polymerase Screening—The Assay of DNA Polymerase Bypass Activity

Only DNA polymerases which can stall at the abasic site during DNA synthesis are suitable for use in the abLAFD method. Thus, a primer extension assay was designed to test the behavior of the four DNA polymerases (Taq, Phusion, Pfu and KAPA) at abasic sites. 

Three primer sets for the amelogenin (AMEL) locus were designed including a conventional primer set (C-primer), a C-primer with additions of a 7-base GTGTCTT 5′-tail (TC-primer), and a TC-primer with an abasic site inserted between the conventional primer and the 7-base GTGTCTT 5′-tail (TaC-primer, Table 1). The 5′-tail is a non-template addition which has been proved to promote full adenylation of PCR products amplified with Taq [23]. All the forward primers were fluorescently labeled using 6-FAM dye. Each DNA polymerase was tested using these three primers sets and 1 ng DNA 9947A according to the manufacturer’s recommendations. PCR products were detected by capillary electrophoresis on an ABI 3130xl genetic analyzer (Thermo Fisher Scientific, Waltham, MA, USA). One microliter of the products was mixed with 9 μL Hi-Di formamide (Thermo Fisher Scientific, Waltham, MA, USA) and a 0.3 μL GeneScan 500 LIZ Dye Size Standard (Thermo Fisher Scientific, Waltham, MA, USA). All alleles were determined with the GeneMapper ID v3.2 (Thermo Fisher Scientific, Waltham, MA, USA). 

#### 2.2.2. Assessment of the Amplification Efficiency of the abLAFD Method

When the procedure of the abLAFD method with suitable DNA polymerase was built, the amplification efficiency of the abLAFD method was comprehensively assessed by quantifying abLAFD products using an absolute quantitative real-time PCR reaction. 

Firstly, singleplex abLAFD reactions were carried out. The primer set with an abasic site near the 3′ end (ab-primer, Table 1) for the AMEL locus was designed. The abasic sites were set at the 6th nt upstream from the 3′ end of the forward ab-primer and 7th nt upstream from the 3′ end of the reverse ab-primer, rendering amplification to be in linear fashion. The abLAFD reaction was carried out with a Phusion Plus PCR Master Mix and prepared in duplicates. The reaction mixture in a final volume of 10 μL contained 5 μL 2 × Phusion Plus PCR Master Mix, 2 μL 10 μM primer mixture, 0.5 μL input DNA (50 pg human female DNA 9947A), and 2.5 μL nuclease-free water. The cycling conditions were 30 s at 98 °C for the initial activation, followed by 30 cycles of 10 s at 98 °C for the denaturation, 20 s at 60 °C for the annealing, 20 s at 72 °C for the extension, and then 5 min at 72 °C for the final extension, and finally maintained at 4 °C. 

Then, a qPCR was carried out to quantify the abLAFD products using a Q-primer (Table 1). One microliter of the abLAFD products was used as a template DNA in the following quantification analysis. All the qPCR reactions were performed with a QuantiNova SYBR Green PCR kit (Qiagen, Duesseldorf, Germany) on a LightCycler 480 System (Roche, Basel, Switzerland). Q-primers for the quantification bind sequences are internal to the abLAFD products. The reaction mixture in a final volume of 10 μL contained 5 μL 2 × SYBR green PCR master mix, 1.4 μL 10 μM primer mixture, 1 μL template DNA, and 2.6 μL nuclease-free water. The cycling condition was 2 min at 95 °C for the initial activation followed by 60 cycles of 5 s at 95 °C for denaturation, and 10 s at 60 °C for annealing and extension. A dilution series of 9947A (1 ng, 300 pg, 100 pg, 50 pg and 30 pg) were prepared and quantified in parallel to generate a standard calibration curve for C_T_ values versus the template amount, which was subsequently used to determine the actual concentration of abLAFD products. Melting curve analysis was also performed to verify the specificity of qPCR products from 65 to 97 °C with the rate of 0.5 °C/s. All the qPCR reactions were run in duplicate. 

### 2.3. Application of abLAFD Method to STR Typing

To demonstrate the use of the abLAFD method in the LT-DNA analysis, a two-step procedure was established by coupling the abLAFD method to conventional STR typing. The abLAFD method contained a multiplex linear amplification reaction and STR typing was performed with the PowerPlex 21 System (PP21, Promega, Madison, WI, USA). 

As for the abLAFD method, primer sets with an abasic site at the 3′ end for five STR loci (D16S539, D18S51, D2S1338, CSF1PO, and Penta D) were pooled together to perform a multiplex amplification (Table S1). These products ranged in size from 289 bp to 569 bp on reference sequences and were long enough to provide primer binding sites for following the PP21 detection. The multiplex abLAFD reactions were performed with 50 pg and 25 pg DNA 9947A in a final volume of 20 μL containing 10 μL 2 × Phusion Plus PCR Master Mix, 8.5 μL 10 μM primer mixture, 0.5 μL input DNA, and 1 μL nuclease-free water. The cycling conditions were 30 s at 98 °C for the initial activation, followed by 30 cycles of 10 s at 98 °C for the denaturation, 20 s at 60 °C for the annealing, 20 s at 72 °C for the extension, and 5 min at 72 °C for the final extension, finally maintained at 4 °C. The experiment was repeated eight times for each DNA input.

Then, 1 μL of products from each abLAFD reaction was used as a template DNA for the following PP21 detection. For comparison purposes, reference STR profiles from 50 pg and 25 pg 9947A were also obtained. All the PP21 reactions in the 12.5 μL final volume were performed according to the manufacturer’s recommendation. The detection of PP21 products was performed on an ABI 3130xl Genetic Analyzer and alleles were determined with the GeneMapper ID v3.2 (Thermo Fisher Scientific, Waltham, MA, USA). 

### 2.4. Data Analysis

To investigate the performance of the abLAFD method on STR typing for the LT-DNA, the allelic drop-out (ADO), allelic drop-in (ADI), allelic peak height (APH), stutter ratio and heterozygote balance (Hb) of STR profiles prepared by the abLAFD method were evaluated and compared with reference to the STR profiles. The APH was determined by the mean peak height of two alleles at heterozygous loci and the halved peak height of the allele at homozygous loci. The Hb was calculated as dividing the peak height of an allele with a lower relative fluorescence unit (RFU) value by the peak height of an allele with a higher RFU value. The stutter ratio was determined by dividing the height of an artificial peak (usually one repeat unit shorter than the true allele) by the peak height of the true allele. The frequencies of the ADO and ADI were obtained by direct counting. The difference in the ADO between abLAFD profiles and reference profiles was assessed by the chi-square test. And the differences in the APH, Hb and stutter ratio between abLAFD profiles and reference profiles were assessed by the unpaired Mann–Whitney U test.

## 3. Results

### 3.1. The Assay of Bypass Activity for DNA Polymerases

**Figure 2:**
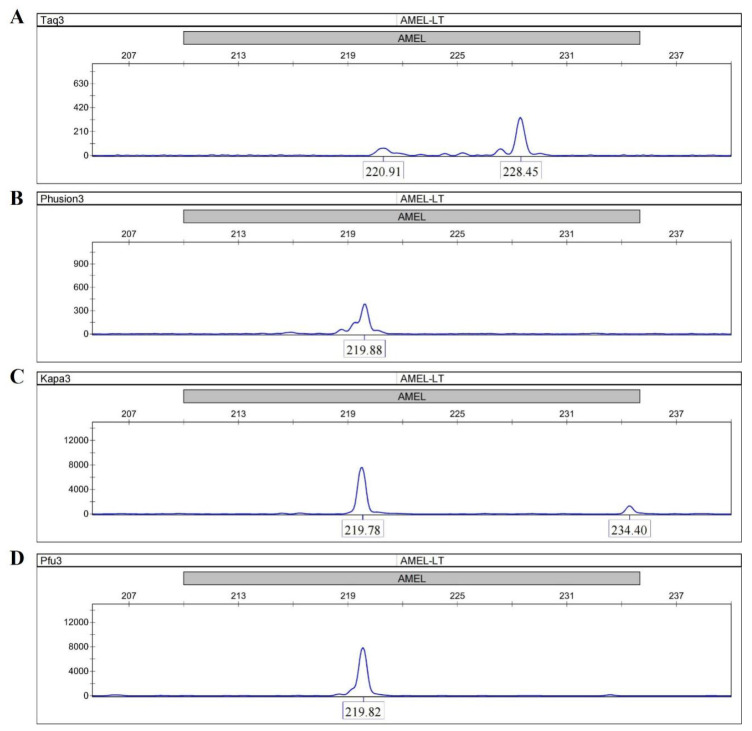
Extensions of TaC primers by the Phusion, Taq, KAPA and Pfu DNA polymerases. (**A**) Taq; (**B**) Phusion; (**C**) KAPA; (**D**) Pfu. Taq gives a main peak at 228.45 bp (full-length) whereas the main peaks in the electropherograms of Phusion, KAPA and Pfu are observed at 219.88 bp, 219.78 bp, and 219.82 bp. The site-specific termination of primer extension can only be successfully achieved in reactions using the three Family B DNA polymerases.

A primer extension assay was performed to investigate the bypass activity of four DNA polymerases (Taq, Phusion, KAPA and Pfu) by using the AMEL locus from female genomic DNA. All the four DNA polymerases were able to amplify full-length sequences with C primers and TC primers (Figures S1 and S2). The peaks in Taq electropherograms were consistently ~1 bp longer than the peaks in other electropherograms due to the adenylation activity of Taq. When TaC primers with abasic sites near the 5′ end were used, distinguishable peak patterns were clearly displayed between the Taq electropherogram and the other three electropherograms. Taq gave a peak at 228.45 bp (full-length), which was 6-fold higher than the expected peak at 220.91 bp (Figure 2A), whereas the main peaks in the electropherograms of Phusion, KAPA and Pfu were observed at 219.88 bp, 219.78 bp, and 219.82 bp (Figure 2B–D). These results suggest that the termination of the primer extension was only successfully achieved in reactions using the three Family B DNA polymerases. In addition, the termination was site-specific, and the three DNA polymerases all stalled precisely opposite the abasic site according to comparisons of product lengths among different primer sets. 

### 3.2. Amplification Efficiency of the abLAFD Method

**Figure 3:**
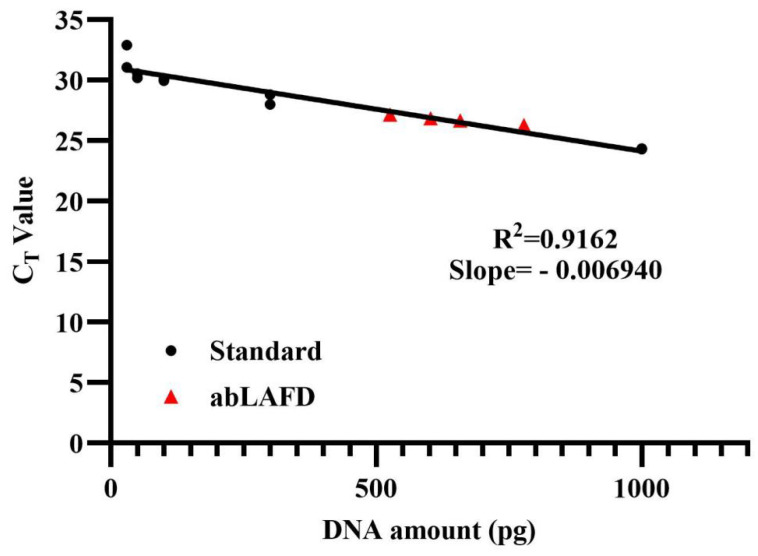
The calibration curve obtained from amplifications of control DNA samples. A dilution series of 9947A (1 ng, 300 pg, 100 pg, 50 pg and 30 pg) were prepared and tested in duplicate as standard samples to generate a calibration curve. The abLAFD reaction was prepared in duplicates and then the abLAFD products from each tube was quantified twice. Standard samples are denoted in black dots and the four abLAFD product samples are denoted in red triangles.

The amplification efficiency of the abLAFD method was estimated by quantifying the abLAFD products amplified from 50 pg DNA. As the abLAFD reaction was in a final volume of 10 μL, 1 μL of abLAFD products was equivalently generated from 5 pg original DNA. The C_T_ values for all samples were shown in Table S2, and the mean C_T_ value for abLAFD product samples was 25.73. Based on the standard calibration curve for C_T_ values (Figure 3), the average copy number of AMEL locus present in 1 μL abLAFD products is equivalent to that present in 650 pg genomic DNA, demonstrating a ~130-fold increase in target DNA by the abLAFD method. A melting curve analysis was also performed in this study (Figure S3). Single peaks at around 77 °C demonstrated the high specificity of the qPCR reactions. 

### 3.3. Evaluation of the Performance of abLAFD Method in STR Typing 

Multiplex abLAFD reactions of five STRs were carried out with 50 pg and 25 pg DNA. Then 1/20 abLAFD products were used as templates in PP21 detection. Representative abLAFD profiles and reference profiles for each DNA input are shown in Figure S4. The ADO, ADI, APH, stutter ratio and Hb were investigated. 

**Figure 4:**
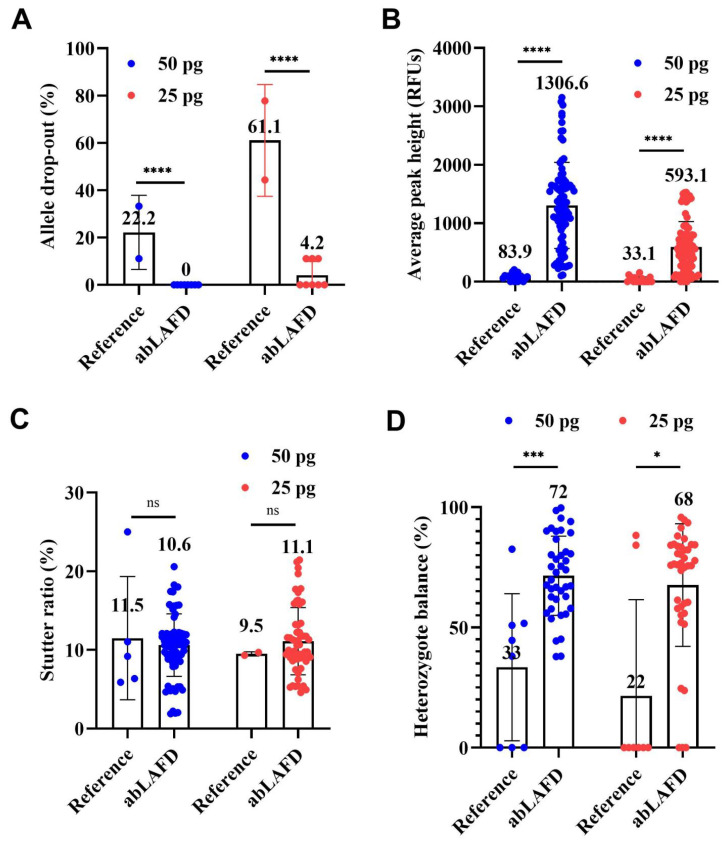
Comparisons among reference profiles and abLAFD profiles amplified from 50 pg DNA and 25 pg DNA. A peak amplitude threshold of 50 RFUs was set for allele calling. The mean value of each dataset was shown above each bar. The threshold significance level was set at 0.05 for each test. The symbols of “****” (or “***”), “*”, and “ns” can be defined as “extremely significant”, “significant”, and “not significant”, respectively. (**A**) The average rate of allele drop-out. It was obtained by direct counting. Significant differences were observed between abLAFD profiles and reference profiles for both 50 pg DNA (*p* < 0.0001) and 25 pg DNA (*p* < 0.0001). (**B**) The allele peak height. It was determined by the mean peak height of two alleles at heterozygous loci and halved peak height of the allele at homozygous loci. Alleles that failed to be called were recorded as 0 RFUs. Significant differences were observed for both 50 pg DNA (*p* < 0.0001) and 25 pg DNA (*p* < 0.0001). (**C**) Stutter ratio. The stutter ratio was determined by dividing the height of an artificial peak (usually one repeat unit shorter than the true allele) by the peak height of the true allele. No significant difference was observed for either 50 pg DNA (*p* = 0.6040) or 25 pg DNA (*p* = 0.7306). (**D**) The heterozygote balance. It was calculated by dividing the peak height of an allele with a lower relative fluorescence unit (RFU) value by the peak height of an allele with a higher RFU value. When one or both of the alleles at a heterozygous locus failed to be detected, the Hb was recorded as 0. Significant differences were observed for both 50 pg DNA (*p* = 0.0006) and 25 pg DNA (*p* = 0.0183).

The average rates of ADO in all STR profiles are shown in Figure 4A. ADO occurred in reference profiles with an average rate of 22.2% for 50 pg DNA and 61.1% for 25 pg DNA (Figure 4A). In comparison, complete results were obtained in all eight abLAFD profiles from 50 pg DNA and five of the eight abLAFD profiles from 25 pg DNA. The average rate of ADO was 0 for 50 pg DNA and 4.2% for 25 pg DNA (Figure 4A), which suggested that the abLAFD method has great capacity for recovering alleles. A chi-square test was performed for the comparison. Significant differences were observed between abLAFD profiles and reference profiles for both 50 pg DNA (*p* < 0.0001) and 25 pg DNA (*p* < 0.0001). No ADI was observed in any profile.

With 50 pg DNA, the APH was 83.9 ± 60.9 RFUs in reference profiles (Figure 4B and Figure S4) and increased by ~15-fold to 1306.6 ± 736.6 RFUs in abLAFD profiles (Figure 4B and Figure S4). When the DNA amount reduced to 25 pg, the APH of the reference profiles fell to 33.1 ± 48.2 RFUs and the abLAFD method resulted in an over-18-fold increase to 593.1 ± 434.3 RFUs. The high level of the standard deviations may be due to the unequal sampling of alleles or amplification bias in PP21 reactions. The unpaired Mann–Whitney U test was performed for the comparison. Significant differences were observed between abLAFD profiles and reference profiles for both 50 pg DNA (*p* < 0.0001) and 25 pg DNA (*p* < 0.0001). For individual loci, the maximum increase in APH was observed in D16S539 (~20 fold) while the minimum was observed in Penta D (~7 fold). 

The stutter ratio ranged from 0.06 to 0.25 with an average value of 0.11 for 50 pg DNA and from 0.09 to 0.10 with an average value of 0.10 for 25 pg DNA when routine STR typing was performed (Figure 4C). In comparison, the average stutter ratio was 0.11 (from 0.02 to 0.21) and 0.11 (from 0.05 to 0.21) for 50 pg and 25 pg DNA samples, respectively, when the abLAFD method was coupled to STR typing (Figure 4C). No significant difference was observed for either 50 pg DNA (*p* = 0.6040) or 25 pg DNA (*p* = 0.7306), which suggests a comparable stutter ratio between procedures with or without the additional step of an abLAFD reaction in forensic STR typing. 

In general, the abLAFD method was able to significantly improve the overall Hb with the increase in the success rate for a correct allele call at heterozygous loci (Figure 4D). The average Hb increased to 72% and 68% for 50 pg and 25 pg DNA samples, respectively, when the abLAFD method was coupled to STR typing, suggesting that each allele at one locus identically accesses the amplification. The unpaired Mann–Whitney U test was performed for the comparison. Significant differences were observed between abLAFD profiles and reference profiles for both 50 pg DNA (*p* = 0.0006) and 25 pg DNA (*p* = 0.0183). 

## 4. Discussion

The faithful preamplification method can allow the detection of very low levels of DNA for traditional STR testing. In this study, we developed the abLAFD method in which the original DNA and its primary amplification products are used as the replication template in each PCR cycle, which should not extend the replication errors during PCR amplification. Our results presented a comparable stutter ratio between reference profiles and abLAFD profiles, which suggested a faithful amplification pattern during the abLAFD method. 

The abLAFD method requires a complete blocking on the extension of the nascent DNA strand at the abasic site. In this study, DNA polymerases from Family B showed the lack of bypass activity and could stall at the abasic site (Figure 2), which may be attributable to the proofreading 3′-5′ exonuclease activity based on available evidence [24,25,26,27,28]. Although a more than 400-fold increase in starting targets was expected under 30 PCR cycles, our results from the qPCR analysis showed about a 130-fold increase in tested AMEL targets, which was consistent with results from the amplification of five STRs. Since all primer sets without an abasic site were validated before any further use, the relatively lower amplification efficiency may be in that the abasic site resulted in a loose combination of primers with template strands to lower the primed efficiency. Further, a low increased fold of targets also suggested a linear amplification fashion, which implied an efficient blocking of extension by the abasic site in the primary nascent strand to result in the absence of a primer binding sequence. 

In this study, the abasic site was designed 5–9 nt away from the 3′ end of primers and could be introduced into primary nascent strands to change the exponential amplification into a linear fashion. In previous studies, a mismatch was commonly added at the 3rd nt of primers at the 3′ end for allele-specific SNP typing [29,30]. Therefore, it is still permissible to optimize the location of the abasic site in primers to improve the amplification efficiency. It should be noted that an abasic site near the 5′ end of primers allows the generation of a primer binding sequence in nascent strands to form an exponential amplification under the annealing temperature (Figure 2). In fact, since this method is similar to the nested PCR in procedure, the specificity can be ensured in the second PCR amplification, i.e., the conventional STR typing. Thus, when a more complex multiplex abLAFD system is developed, a lower annealing temperature (e.g., 58 °C) can be applied in the abLAFD reaction to increase primer-binding efficiency without any concern over the priming specificity. Additionally, a semi-abLAFD method, that applies one primer with an abasic site together with a regular primer for amplification, can employ original DNA, primary and secondary nascent strands as templates to increase the PCR yield. 

The nested PCR applies external primers for exponential amplification, which can result in the accumulation of slippage mutation and bring increased PCR artifacts to the following STR typing [7]. In contrast, the linear amplification provided high fidelity for the amplification of original DNA. Meanwhile, the abLAFD method may allow identical access of all alleles at single loci to the amplification. Our results showed that the abLAFD method coupled with conventional STR typing increased the allele peak height and heterozygote balance together with comparable stutter ratios. In fact, the abLAFD method theoretically increases the N(N+1)/2 fold of the starting DNA template when using N PCR cycles. The increase in PCR cycles should not raise the accumulation of amplification errors, since the abLAFD method only employs the original DNA and its primary nascent products as templates. Therefore, combining the abLAFD method with conventional STR typing can provide reliable STR profiles for forensic LT-DNA analysis.

As this preliminary study was intended to investigate the feasibility and potential of the abLAFD method, the external primers used in the multiplex abLAFD reactions were designed specifically for the PP21 system. In fact, a much bigger step can be developed in a future study. A universal multiplex abLAFD system, which contains a set of CODIS STR loci to provide sufficient discrimination power for forensic use, can be developed by intentionally extending both the up- and downstream flanking region of each locus to around 300 bp. Consequently, the system can be coupled to some commercial kits in the case where various detection systems are applied in different labs.

In this study, we identified three DNA polymerases which lack bypass activity. By employing one of these DNA polymerases and primers with abasic sites near the 3′ end, we developed a novel linear amplification method abLAFD. When the abLAFD method was additionally performed before routine PP21 STR tests with 50 pg DNA and 25 pg DNA, significantly recovered alleles, increased average peak height and a heterozygote balance with a comparable stutter ratio were observed in STR profiles. These results strongly support the theory that the abLAFD method can be a promising strategy coupled to STR typing for forensic LT-DNA analysis.

## Figures and Tables

**Table 1:** Overview of primer sets for detection of AMEL locus in the primer extension assay and the quantification assay.

Primer	Primer Sequence (5′–3′)
C-primer	F: FAM-CACCTCATCCTGGGCACCC R: GGCTTGAGGCCAACCATCAG
TC-primer	F: FAM-GTGTCTTCACCTCATCCTGGGCACCC R: GTGTCTTGGCTTGAGGCCAACCATCAG
TaC-primer	F: FAM-GTGTCTT/idSp/CACCTCATCCTGGGCACCC R: GTGTCTT/idSp/GGCTTGAGGCCAACCATCAG
ab-primer	F: CTACCACCTCATCCTGGGC/idSp/CCCTG R: GAGGACAGACTGAGTCAGAG/idSp/GGCCAG
Q-primer	F: CCTGGGCTCTGTAAAGAATAGTG R: CAGAGCTTAAACTGGGAAGCTG

The location of the abasic site generated by the dSpacer modification is denoted as /idSp/ in the primer sequence. Forward primers designed for the primer extension assay are fluorescently labeled using 6-FAM dye. All the primer sets facilitate exponential amplification, except for the ab-primer set.

## Data Availability

The dataset is available from the corresponding author on reasonable request.

## Supplementary Materials

The following supporting information can be downloaded at: https://www.mdpi.com/article/10.3390/genes13081386/s1, Figure S1: Extensions of C-primer by the Phusion, Taq, KAPA and Pfu DNA polymerases; Figure S2: Extensions of TC-primer by the Phusion, Taq, KAPA and Pfu DNA polymerases; Figure S3: Melting data of standard DNA samples and abLAFD samples; Figure S4: Representative abLAFD profiles and reference profiles amplified from 50 pg DNA and 25 pg DNA; Table S1: Overview of primer sets for the five STRs in the multiplex abLAFD reactions; Table S2: The C_T_ value and concentration for all reactions in the absolute quantitative real-time PCR.
