# Supplementary material for: Investigation of Linear Amplification Using Abasic Site-Containing Primers Coupled to Routine STR Typing for LT-DNA Analysis
Source: Genes (Basel). 2022 Aug 4;13(8):1386. doi: 10.3390/genes13081386 (PMC9407227; doi:10.3390/genes13081386)
Supplement: Supplementary file 1 [file genes-13-01386-s001.zip › genes-1833921-supplementary.pdf]

Supplementary Information

Investigation of linear amplification using abasic site-containing primers coupled to routine STR typing for LT-DNA analysis

Xiaoqin Qian[a], Zhimin Li[a], Zhihan Zhou[a], Jinglei Qian[a], Yining Yao[a], Chengchen Shao[a], Qiqun Tang[b], Jianhui Xie [a]

[a] Department of Forensic Medicine, School of Basic Medical Sciences, Fudan University, Shanghai, 200032, China

[b] Department of Biochemistry and Molecular Biology, School of Basic Medical Sciences, Fudan University, Shanghai, 200032, China

***Corresponding author:**

Jianhui Xie, Ph. D.

Department of Forensic Medicine, School of Basic Medical Sciences, Fudan University, Shanghai, 200032, China

Address: 138 Yixueyuan Road, Shanghai, 200032, China

Tel: +86-021-54237403

Email: jhxie@fudan.edu.cn

Supplementary Tables

Table S1: Overview of primer sets for the five STRs in the multiplex abLAFD reactions.

Locus	Primer sequence (5′ – 3′)	Final concentration	Secondary product size (nt)
D16S539	F: GTGTACAAGTGCCAGA/idSp/GCTCG	0.14 µM	291
	R: GTAAAACAGCCTACAGAG/idSp/GATTCC		289
D18S51	F: TGTAGTCTCAGCTACTT/idSp/CAGGGC	2.08 µM	362
	R: AAATAACAAACCC/idSp/ACTACCAGC		366
D2S1338	F: GTGACATCAATACGTTCATTTCTT/idSp/CTAGCAC	1.11 µM	482
	R: CCGGAATCAGTACTATATTGG/idSp/GAGCTG		485
CSF1PO	F: CACTGGCCATCTTCAGC/idSp/CATTCTC	0.56 µM	569
	R: GGGAGGAACATATGCAAGGC/idSp/CAAAGG		566
Penta D	F: CATGGTGAGGCTGAAGTAGGATC/idSp/CTTG	1.11 µM	550
	R: CTCTCAAAGTGCTGGGATTA/idSp/CATCGC		553

/idSp/: an abasic site; F: forward primer; R: reverse primer.

Table S2: The C_T value and concentration for all reactions in the absolute quantitative real-time PCR.

Sample	Type	C_T	Concentration (ng)
1ng	Standard	24.30	
1ng	Standard	24.35	
300pg	Standard	28.80	
300pg	Standard	27.99	
100pg	Standard	30.08	
100pg	Standard	29.94	
50pg	Standard	30.19	
50pg	Standard	30.53	
30pg	Standard	31.06	
30pg	Standard	32.89	
abLAFD product sample 1	Unknown	25.64	0.648
abLAFD product sample 2	Unknown	25.27	0.768
abLAFD product sample 3	Unknown	25.84	0.592
abLAFD product sample 4	Unknown	26.15	0.516

Supplementary Figures

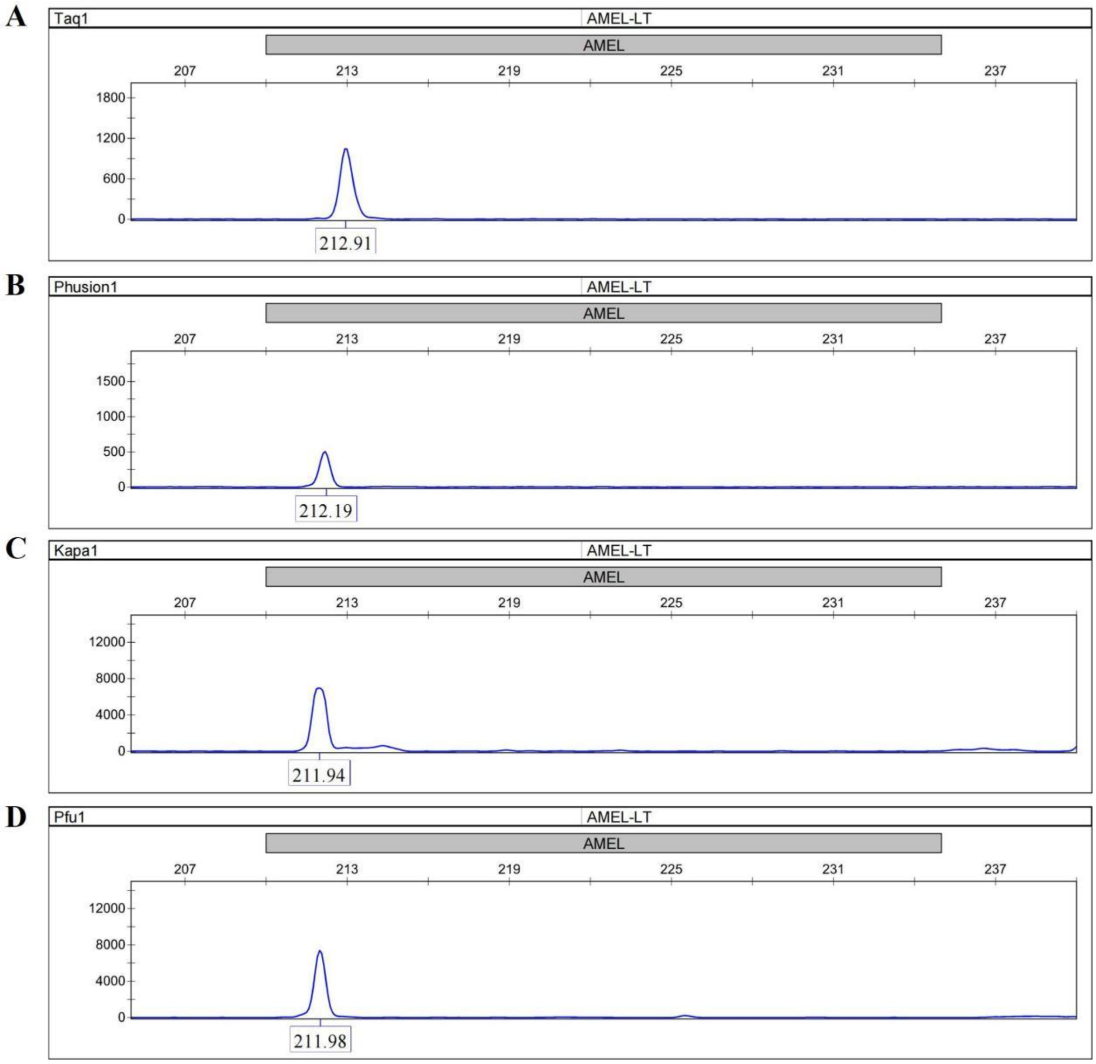

Figure S1: Extensions of C-primer by the Phusion, Taq, KAPA and Pfu DNA polymerases. (A): Taq; (B): Phusion; (C): KAPA; (D): Pfu. All the four DNA polymerases could amplify full-length sequences with C-primers. The peak in the Taq electropherogram was ~1 bp longer in length than peaks in other electropherograms due to the adenylation activity of Taq.

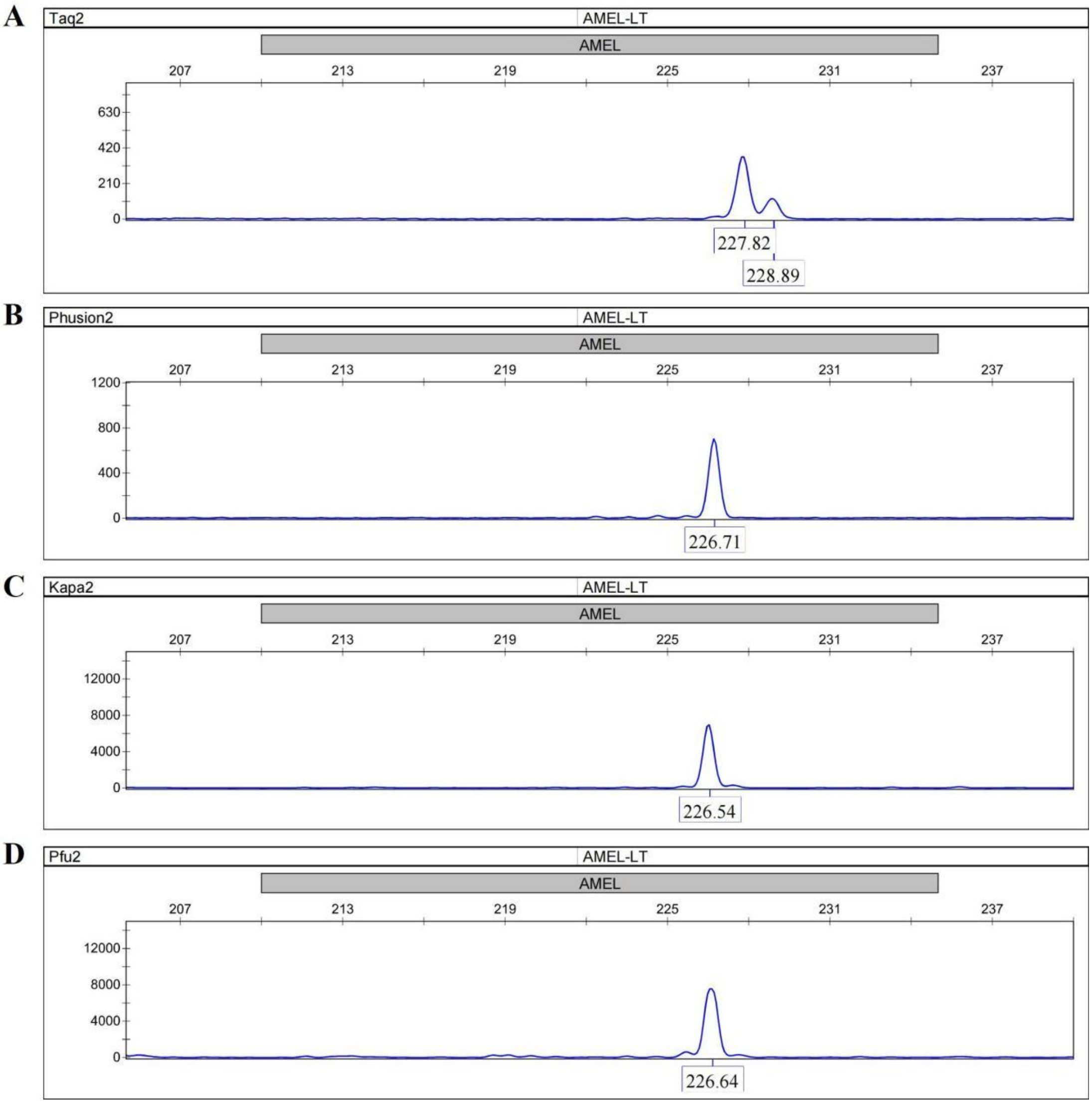

Figure S2: Extensions of TC-primer by the Phusion, Taq, KAPA and Pfu DNA polymerases. (A): Taq; (B): Phusion; (C): KAPA; (D): Pfu. All the four DNA polymerases could amplify full-length sequences with TC-primers. The main peak in the Taq electropherogram was ~1 bp longer in length than peaks in other electropherograms due to the adenylation activity of Taq.

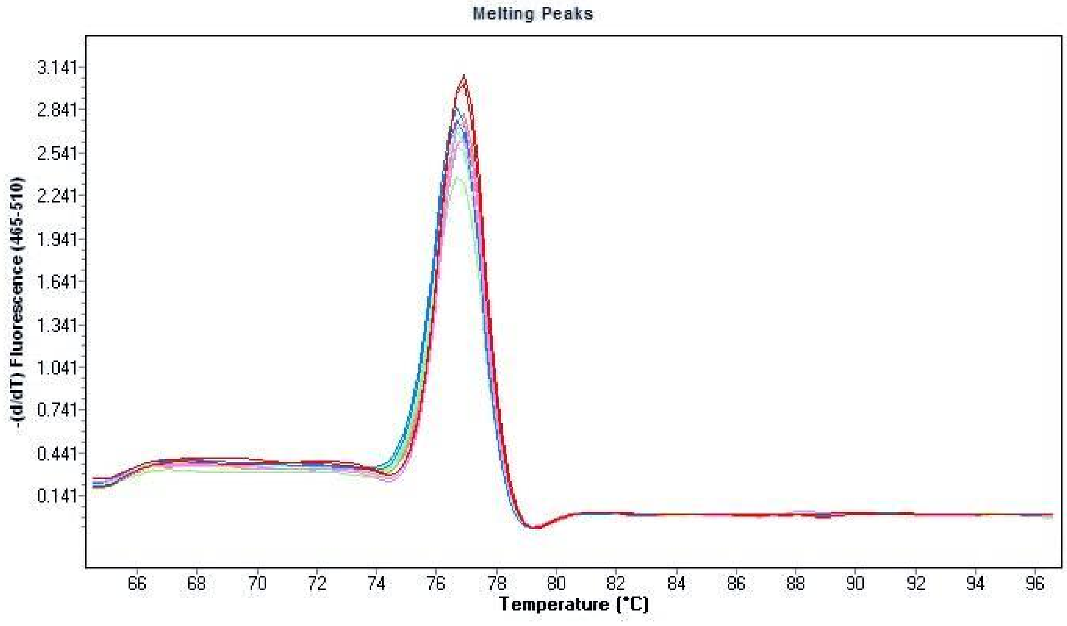

Figure S3: Melting data of standard DNA samples and abLAFD samples.

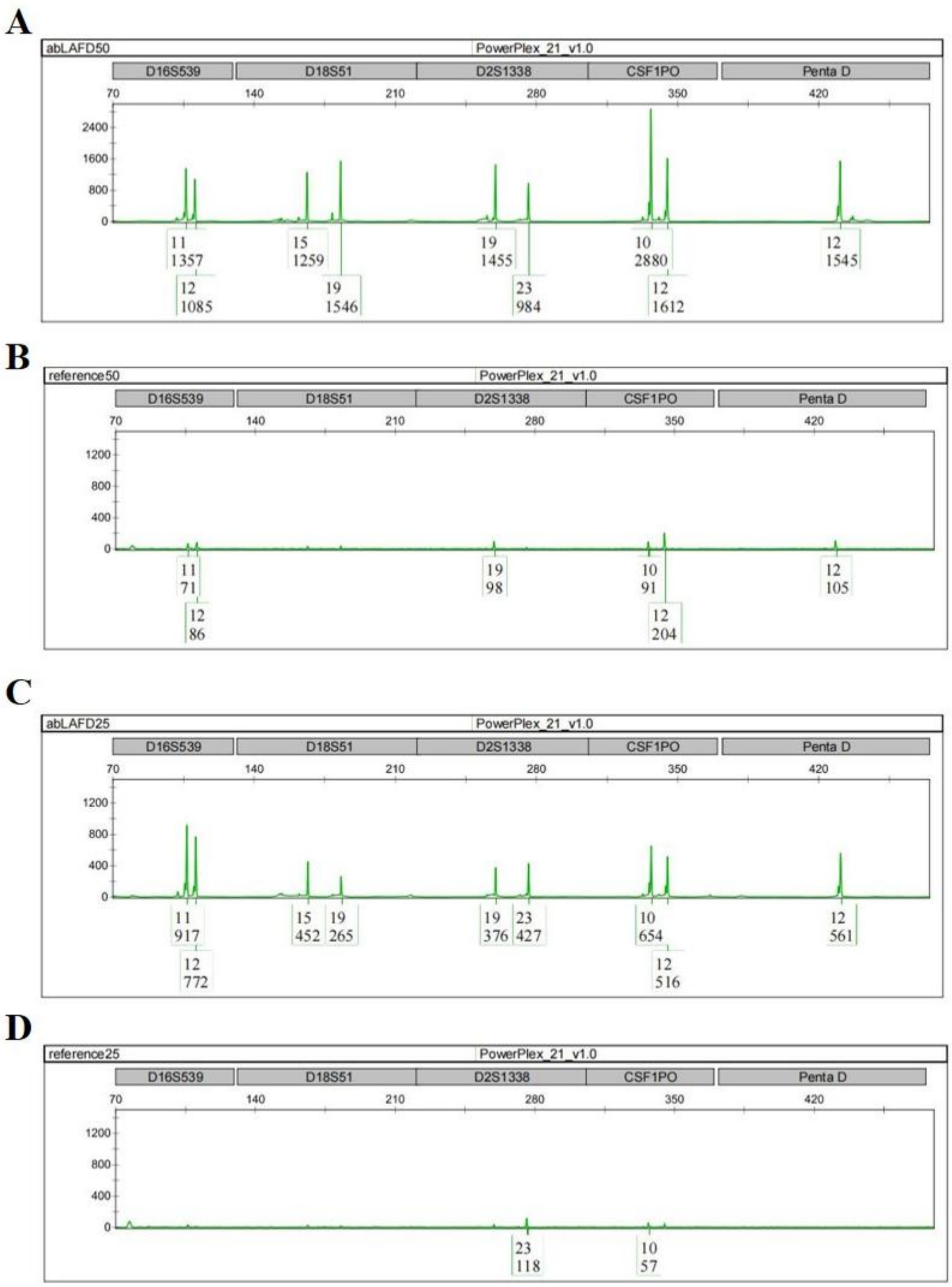

Figure S4: Representative abLAFD profiles and reference profiles amplified from 50 pg DNA and 25 pg DNA. (A): An abLAFD profile amplified from 50 pg DNA; (B): A reference profile amplified from 50 pg DNA; (C): An abLAFD profile amplified from 25 pg DNA; (D): A reference profile amplified from 25 pg DNA. The allelic name and height are shown under each peak. When a threshold of 50 RFUs is applied for allele calling in data analysis, the average success rate for a correct allele call is 100% and 95.8% for abLAFD profiles amplified from 50 pg DNA and 25 pg DNA, but only 77.8% and 38.9% for reference profiles. Compared to reference profiles, the APH of abLAFD profiles amplified from 50 pg DNA and 25 pg DNA is increased by ~15-fold to 1306.6 ± 736.6 RFUs and by ~18-fold increase to 593.1 ± 434.3 RFUs, respectively.
